# The transcription factor RUNX2 regulates receptor tyrosine kinase expression in melanoma

**DOI:** 10.18632/oncotarget.8822

**Published:** 2016-04-18

**Authors:** Rajeev K. Boregowda, Daniel J. Medina, Elke Markert, Michael A. Bryan, Wenjin Chen, Suzie Chen, Anna Rabkin, Michael J. Vido, Samuel I. Gunderson, Marina Chekmareva, David J. Foran, Ahmed Lasfar, James S. Goydos, Karine A. Cohen-Solal

**Affiliations:** ^1^ Division of Medical Oncology, Department of Medicine, Robert Wood Johnson Medical School, Rutgers Cancer Institute of New Jersey, Rutgers, The State University of New Jersey, New Brunswick, NJ 08903, USA; ^2^ Cancer Research UK Beatson Institute, Glasgow, G61 1BD Scotland, UK; ^3^ Center for Biomedical Imaging & Informatics, Rutgers Cancer Institute of New Jersey, New Brunswick, NJ 08903, USA; ^4^ Department of Pathology and Laboratory Medicine, Robert Wood Johnson University Hospital, Rutgers Cancer Institute of New Jersey, Rutgers, The State University of New Jersey, New Brunswick, NJ 08901, USA; ^5^ Department of Chemical Biology, Susan Lehman Cullman Laboratory for Cancer Research, Ernest Mario School of Pharmacy, Rutgers, the State University of New Jersey, New Brunswick, NJ 08903, USA; ^6^ Department of Cancer Biology and Sidney Kimmel Cancer Center, Thomas Jefferson University, Philadelphia, PA 19107, USA; ^7^ Department of Molecular Biology and Biochemistry, Rutgers University, Piscataway, NJ 08854, USA; ^8^ Department of Pharmacology and Toxicology, Ernest Mario School of Pharmacy, Rutgers, The State University of New Jersey, Piscataway, NJ 08854, USA; ^9^ Rutgers Cancer Institute of New Jersey, New Brunswick, NJ 08903, USA; ^10^ Division of Surgical Oncology, Department of Surgery, Robert Wood Johnson Medical School, Rutgers Cancer Institute of New Jersey, Rutgers, the State University of New Jersey, New Brunswick, NJ 08901, USA; ^11^ Section of Surgical Oncology Research, Department of Surgery, Robert Wood Johnson Medical School, Rutgers Cancer Institute of New Jersey, Rutgers, The State University of New Jersey, New Brunswick, NJ 08901, USA

**Keywords:** melanoma, transcription factor, RUNX2, receptor tyrosine kinase, resistance to targeted therapy

## Abstract

Receptor tyrosine kinases-based autocrine loops largely contribute to activate the MAPK and PI3K/AKT pathways in melanoma. However, the molecular mechanisms involved in generating these autocrine loops are still largely unknown. In the present study, we examine the role of the transcription factor RUNX2 in the regulation of receptor tyrosine kinase (RTK) expression in melanoma. We have demonstrated that RUNX2-deficient melanoma cells display a significant decrease in three receptor tyrosine kinases, EGFR, IGF-1R and PDGFRβ. In addition, we found co-expression of RUNX2 and another RTK, AXL, in both melanoma cells and melanoma patient samples. We observed a decrease in phosphoAKT2 (S474) and phosphoAKT (T308) levels when RUNX2 knock down resulted in significant RTK down regulation. Finally, we showed a dramatic up regulation of RUNX2 expression with concomitant up-regulation of EGFR, IGF-1R and AXL in melanoma cells resistant to the BRAF V600E inhibitor PLX4720. Taken together, our results strongly suggest that RUNX2 might be a key player in RTK-based autocrine loops and a mediator of resistance to BRAF V600E inhibitors involving RTK up regulation in melanoma.

## INTRODUCTION

The RUNX (Runt-related transcription factor) family is comprised of three closely related transcription factors, RUNX1, RUNX2 and RUNX3. These genes are defined by a highly conserved 128 amino acid DNA binding/protein-protein interaction domain called the Runt-homology domain [1]. RUNX2 is a major determinant of osteoblast differentiation and regulates chondrocyte proliferation, differentiation and hypertrophy during endochondral bone formation [2–5]. In addition, RUNX2 regulates the expression of genes closely associated with tumor progression, invasion and metastasis [1, 6–9] and has pro-angiogenic effects [10–13]. We were the first to demonstrate that RUNX2 was overexpressed in human melanoma cells as compared with normal human melanocytes and that RUNX2 deficiency inhibited cell growth, migration and invasion of human melanoma cells. In addition, we showed that decreased RUNX2 expression was associated with a reduction in the expression of FAK [14], implicated in cell migration [9, 15] and melanoma metastasis [16–18].

Up-regulation of the expression of EGF-R, PDGFRβ, AXL and IGF-1R in melanoma cells that have developed resistance to BRAF V600E targeted therapy [19–25] results in reactivation of the MAPK and the PI3K/AKT pathways. These pathways are crucial not only for tumor cell survival and proliferation, but also for continued tumor cell migration and invasion [26–31]. However, the mechanisms contributing to high levels of these RTKs in melanomas have not been elucidated either in the context of progressing tumors or in acquired resistance to BRAF V600E inhibitors.

An early analysis of the expression of growth factors and their receptors in melanomas showed that EGF was overexpressed as compared to melanocytes while EGFR was expressed in both melanomas and melanocytes. This suggests a role for EGF-EGFR-based autocrine signaling in melanoma cells [32]. In favor of this role, treatment with cetuximab, a monoclonal antibody targeting the extracellular domain of EGFR, reduced the invasive ability of melanoma cells. Furthermore, in 114 patients with primary cutaneous melanomas, EGFR expression was found to be associated with metastatic spread to sentinel lymph nodes and the presence of EGFR polysomy was correlated with thicker primary tumors [33]. FGF-FGFR -based autocrine signaling was also suggested by overexpression of FGF2, FGF5 and FGF8 and different isoforms of the FGF receptors (FGFR1-4) in melanoma cells. In addition, it has been shown that stimulation of FGF receptors enhances the motility of melanoma cells [32, 34].

IGF1R activation was reported in a panel of 25 melanoma cell lines derived from primary and metastatic lesions [35] and a comprehensive analysis of RTK activation in human melanoma samples and cell lines identified autocrine signaling through IGF-1R [36]. Disruption of the direct interaction between Focal Adhesion Kinase (FAK) and IGF-1R by the small molecule compound INT2-31 inhibited melanoma xenograft growth in association with reduced levels of pAKT (S473) [37]. A study on uveal melanoma supports the role of exogenous and endogenous IGF-1 and their interaction with IFG-1R in the development of metastases in the liver, which is a major site for IGF-1 production and the predominant metastatic site in 70-90% of uveal melanoma [38]. The contribution of IGF-1 to melanoma cell migration was shown to require the activation of PI3K by IGF-1R [39].

AXL is activated in melanomas [35] and engaged in an autocrine loop due to endogenous production of its ligand Gas6 [40]. In addition, AXL promotes the pro-migratory and pro-invasive behavior of melanoma cells [35, 40] and resistance to the BRAF V600E inhibitor PLX4720 [20]. These findings are reinforced in a more recent study, which demonstrated an increased resistance to BRAF and ERK inhibition in melanoma cells expressing low levels of MITF (Microphthalmia-associated transcription factor) and high levels of AXL. This study also showed a reverse correlation between MITF and receptor tyrosine kinases, including AXL [21]. This study confirmed the negative correlation between MITF and AXL in melanoma cells [40, 41]. Since loss of MITF is also accompanied by increased invasiveness [21, 42], it supports previous findings that a high level of AXL expression is associated with a pro-invasive phenotype in melanoma.

The present study was designed to investigate the relationship between RUNX2 and the expression of receptor tyrosine kinases (RTKs) in melanoma. We demonstrated the co-expression of RUNX2 with IGF-1R, EGF-R, PDGFRβ and AXL using ShRNA RUNX2-expressing melanoma cells and showed co-expression of RUNX2 with AXL in human melanoma samples. Most importantly, we demonstrate for the first time that melanoma cells resistant to the BRAF V600E inhibitor PLX4720 had a significant increase in RUNX2 expression associated with an increase in RTKs expression and activation. In addition, melanoma cells with reduced expression of RUNX2 had an increased sensitivity to PLX4720. We suggest that RUNX2 may be a driver of increased receptor-tyrosine kinase (RTK)-based autocrine signaling in melanoma and a key player in resistance to targeted therapies involving up regulation of different RTKs.

## RESULTS

### ShRNA-mediated depletion of RUNX2 reduces RTKs expression

We previously demonstrated that knock down of RUNX2 using RUNX2 shRNA lentiviral expression vectors decreased melanoma cell migration and invasion. These findings suggest a role for RUNX2 in the migration and invasive ability of melanoma cells [14]. In order to define potential mediators of the RUNX2-mediated effects on migration and invasion, we generated 250 μg of proteins from stable 1205LU melanoma cell lines expressing two types of RUNX2 ShRNA; ShRUNX2-2 (targeting RUNX2 3′UTR) and ShRUNX2-3 (targeting the RUNX2 coding sequence) as previously described [14]. We then performed a quantitative analysis of proteins up- or down-regulated in the RUNX2 knocked down 1205LU cells relative to non-silencing ShRNA-expressing 1205LU cells using mass spectrometry. As shown in Table 1, reduced levels of the receptor tyrosine kinases IGF-1R, FGFR1, PDGFRβ, and EGFR were detected in the RUNX2 knocked down 1205LU melanoma cells as compared with the non-silencing (control) ShRNA-expressing 1205LU cells. Because IGF-1R, FGFR1, and EGFR have been shown to play major roles in melanoma progression through autocrine signaling [32, 34, 36], and EGF-R, PDGFRβ, and IGF-1R have been implicated in resistance mechanisms to BRAF V600E targeted therapies [19–22, 24, 25], we were interested in validating these results. Therefore, we compared other melanoma cell lines expressing non-silencing ShRNA, ShRUNX2-2 or ShRUNX2-3 for the expression of these RTKs.

**Table 1:** Proteomics analysis of the ShRNA RUNX2-2- and ShRNA RUNX2-3-expressing 1205LU melanoma cells as compared with non-silencing (NS) control 1205LU expressing cells

Receptor tyrosine kinase (RTK)	ShRUNX2-2 ShRNA/NS ShRNA	ShRUNX2-3 ShRNA/NS ShRNA
IGF-1R	NC	0.83
FGFR1	0.71	0.71
PDGFRβ	0.85	0.83
EGFR	NC	0.81

The protein expression of the listed receptor tyrosine kinases is expressed as ratio between ShRNA RUNX2-2- or ShRNA RUNX2-3-1205LU cells and NS-1205LU cells. PDGFRβ: Platelet-derived growth factor receptor, beta; EGFR: epidermal growth factor receptor; FGFR1: fibroblast growth factor receptor 1; IGF-1R: insulin-like growth factor 1 receptor. NC: No Change.

In addition to these RTKs identified by mass spectrometry, we were also interested in identifying other potential RTKs co-regulated with RUNX2 using patient data. Using the CBio Portal for cancer genomics (http://www.cbioportal.org) [43, 44], we found that the receptor tyrosine kinase AXL was co-expressed with RUNX2 (Pearson's correlation = 0.56) after analysis of 278 skin cutaneous melanomas (TCGA provisional RNASeqV2 RSEM).

**Figure 1:**
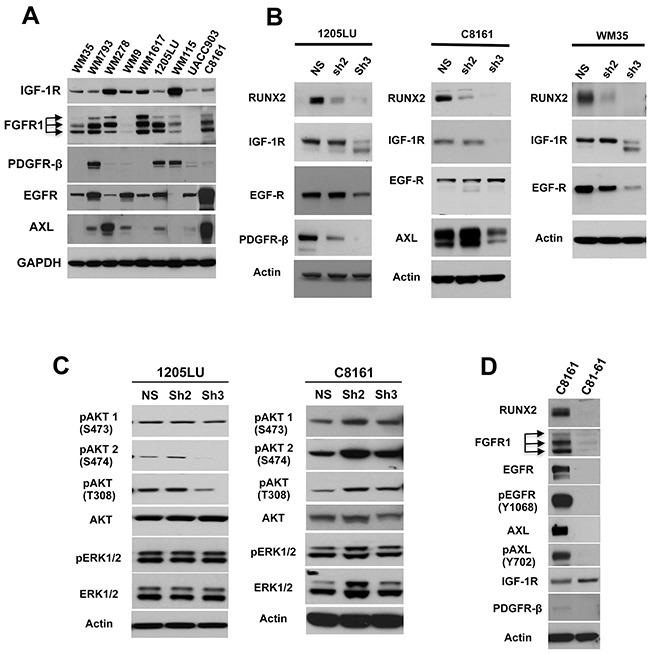
RUNX2 knock down results in reduced expression of RTKs **A.** Lysates from melanoma cell lines were analyzed for the expression of IGF-1R, FGFR1, PDGFRβ, EGFR and AXL. **B.** RUNX2 and RTKs levels in non-silencing ShRNA (NS), ShRUNX2-2 (Sh2) and ShRUNX2-3 (Sh3) stable melanoma cell lines. **C.** Levels of pAKT(T308), pAKT1(S473), pAKT2(S474), AKT, pERK1/2, ERK1/2 and Actin in non-silencing ShRNA (NS), ShRUNX2-2 (Sh2) and ShRUNX2-3 (Sh3) stable melanoma cell lines. **D.** Levels of RUNX2, FGFR1, EGFR, AXL, IGF-1R, PDFGRβ, phosphorylated EGFR (Y1068) and phosphorylated AXL (Y702) in C8161 and C81-61 melanoma cell lines.

To determine which melanoma cell lines to use for validation of the co-expression of RUNX2 and the aforementioned RTKs, we analyzed the expression of these receptors in a panel of melanoma cell lines. As shown in Figure 1A, IGF-1R is expressed by all the melanoma cell lines and FGFR1 is highly expressed in 7 out of the 9 analyzed melanoma cell lines. EGFR is significantly expressed in melanoma cell lines with the exception of one. AXL is expressed in 6 out of the 9 melanoma cell lines tested, and PDGFRβ is expressed at high levels in three cell lines and at lower levels or undetectable in the others. The expression level of the different RTKs seems independent of the stage of progression, when we compare cells derived from vertical growth phase (VGP) melanomas (WM793, WM278 or WM115) or from metastases (WM9, WM16717, 1205LU, C8161 and UACC903). We further chose three cell lines (1205LU, C8161 and WM35) for which RUNX2 knock down using ShRUNX2-2 and ShRUNX2-3 was pronounced to validate the co-expression of RUNX2 and RTKs.

Immunoblot analysis of 1205LU cells expressing non-silencing ShRNA, ShRUNX2-2 or ShRUNX2-3 showed reduced levels of IGF-1R and EGFR only in ShRUNX2-3-expressing 1205LU cells exhibiting the strongest RUNX2 knock down. The expression of PDGFRβ showed a decrease in both ShRUNX2-2- and ShRUNX2-3-expressing 1205LU cells as compared with non-silencing ShRNA-expressing 1205LU control cells (Figure 1B, left panel), in accordance with Table 1. Therefore, three of the four RTKs that we found negatively regulated in the RUNX2-knocked down 1205LU melanoma cells by mass spectrometry were validated by immunoblot analysis. The expression of IGF-1R was reduced in ShRUNX2-3-expressing C8161 cells or ShRUNX2-3-expressing WM35 cells, as compared with non-silencing ShRNA-expressing C8161 control cells or non-silencing ShRNA-expressing WM35 control cells respectively. Thus, as described for 1205LU cells, we observe a decrease in IGF-1R only in cells demonstrating nearly complete RUNX2 knock down (Figure 1B, middle and right panels). Like IGF-1R, AXL expression is only reduced in ShRUNX2-3-expressing C8161 cells as compared with non-silencing ShRNA-expressing C8161 cells (Figure 1B, middle panel). EGFR expression is diminished in WM35 expressing ShRUNX2-2 or ShRUNX2-3 as compared with non-silencing ShRNA-expressing WM35 cells. The strongest reduction of EGFR expression was observed in the ShRUNX2-3-expressing WM35 cells exhibiting almost complete RUNX2 knockdown (Figure 1B right panel). We did not detect changes in the expression of FGFR1 in any of the RUNX2-knocked down melanoma cell lines (data not shown). These results altogether show that RUNX2 knock down has a negative effect on the expression of selected RTKs, with IGF-1R being downregulated in three melanoma cell lines exhibiting RUNX2 knock down and EGFR in two out of three cell lines.

RTKs stimulation leads to activation of the RAS/MAPK and PI3K/AKT pathways [45]. Activation of the RAS/RAF/MEK cascade leads to ERK1 and ERK2 phosphorylation [46] at T202/Y204 and T185/Y187 respectively. After activation of PI3K and subsequent conversion of PIP_2_ to PIP_3_, AKT binds to PIP_3_ at the membrane allowing PDK1 to phosphorylate T308 leading to partial activation of AKT. Phosphorylation of AKT at S473 (AKT1) or S474 (AKT2) stimulates full activity [47]. We were interested in analyzing whether reduction of RTK expression resulted in inhibition of phosphorylation of ERK and AKT in RUNX2 knocked down melanoma cell lines. Since opposing effects of AKT1 and AKT2 were described in breast cancer cell migration, AKT1 suppressing invasion and AKT2 enhancing it [48], we analyzed phosphorylation of AKT1 and AKT2, in addition to T308 in the RUNX2 knocked down melanoma cell lines. As shown in Figure 1C, 1205LU expressing ShRUNX2-3 demonstrated reduction of AKT phosphorylated at T308 as compared with the non-silencing controls. In addition, in 1205LU melanoma cell lines expressing ShRUNX2-3, phosphorylation of AKT2 at S474 was decreased as compared with the non-silencing control-expressing 1205LU cells. No modulation of phosphoAKT1(S473) was detected in 1205LU melanoma cells expressing ShRUNX2-2 or ShRUNX2-3. By contrast, we did not observe any decrease in pAKT(T308), pAKT1(S473) and pAKT2(S474) levels in C8161 ShRUNX2-2 or C8161 ShRUNX2-3 as compared with C8161 expressing the non-silencing control. This result could be explained by the maintained expression of EGFR in C8161 cells expressing ShRUNX2-2 or ShRUNX2-3. EGFR expression in those lines could trigger phosphorylation/activation of AKT, as well as phosphorylation/activation of ERK1/2 as observed in Figure 1C. Furthermore, the reduced expression of RUNX2 and RTKs in 1205LU expressing ShRUNX2-3 did not result in reduced levels of pERK1/2 levels (Figure 1C). 1205LU melanoma cells carry the BRAF V600E activating mutation responsible to maintain an activated MAPK/ERK pathway, reflected in high levels of pERK1/2 [49]. Therefore, our results are in accordance with earlier studies showing that the activation of the MAPK/ERK pathway can be driven only by the BRAF activating mutation, independently of RTKs activation.

To further analyze the co-expression of RUNX2 and RTKs, we examined the expression of RTKs in C81-61 and C8161 melanoma cell lines ([50]. The C81-61 cell line is derived from a Vertical-Growth Phase Melanoma and the C8161 cell line from a metastasis from the same patient. As shown in Figure 1D, C81-61 cells had undetectable levels of RUNX2, in contrast to C8161 cells. In addition, FGFR1, EGFR, and AXL were expressed at high levels in C8161 cells as compared to C81-61 cells (Figure 1D). We are aware that C8161 and C81-61 cell lines might have numerous genetic and epigenetics differences in addition to the differential expression of RUNX2. However, it indicates that AXL might be an important RTK regulated by RUNX2, since we also found decreased levels of this RTK in C8161 expressing ShRUNX2-3, which induces the strongest RUNX2 knock down. In addition, we found high levels of phosphorylated EGFR (Y1068) and phosphorylated AXL (Y702) in C8161 melanoma cells, in contrast to C81-61 melanoma cells. These results suggest that these RTKs are not only up-regulated but also activated in C8161 melanoma cells.

### RUNX2 and AXL co-expression in melanoma samples

**Figure 2:**
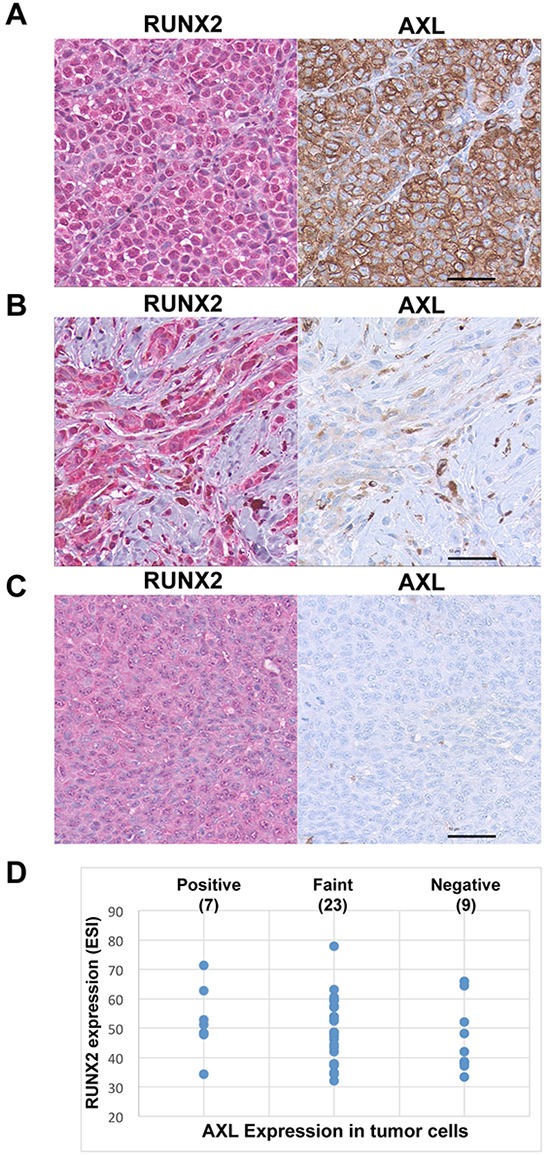
RUNX2 and AXL co-expression in melanoma samples Representative pictures of strong **A.**, faint **B.** and negative **C.** AXL staining in RUNX2-expressing melanoma cores from a melanoma tissue microarray. RUNX2 staining (Chromogenic detection using red) in on the left panel, corresponding microscopic view of AXL staining (Chromogenic detection using DAB, brown) is on the right panel. **D.** Expression levels of RUNX2 and AXL in the 39 RUNX2-expressing melanoma samples analyzed. The Y axis shows quantitative Effective Staining Intensity (ESI; [71]) of RUNX2 in melanoma patient samples. The X axis expresses qualitative analysis of AXL staining defined as strong, faint and negative. The scale bar represents 50 μm.

The results presented in Figure 1 prompted us to analyze the co-expression of RUNX2 and AXL in human melanoma samples. First, analysis of 278 cutaneous melanomas (TCGA provisional RNASeqV2 RSEM) using the CBio Portal for cancer genomics [43, 44] indicated that AXL was co-expressed with RUNX2 (Pearson's correlation = 0.56). To confirm the mRNA expression results, we performed an immunohistochemical analysis of RUNX2 and AXL in the same melanoma tissue microarray (TMA). The slides used for RUNX2 and AXL immunohistochemical staining were two serial sections of the TMA. Figure 2 shows representative pictures of RUNX2 and AXL immunostaining of 39 cores expressing significant levels of RUNX2 and exhibiting strong diffuse (Figure 2A), faint focal (Figure 2B) or no (Figure 2C) AXL staining. Analysis of the 39 cores expressing RUNX2 showed that 7 had strong AXL staining, 23 had faint AXL staining and 9 were negative for AXL (Figure 2D). These results suggest the co-expression of RUNX2 and AXL proteins in a significant number of human melanoma lesions.

### U1 Adaptor oligonucleotides targeting RUNX2 inhibit RUNX2 and RTK expression

**Figure 3:**
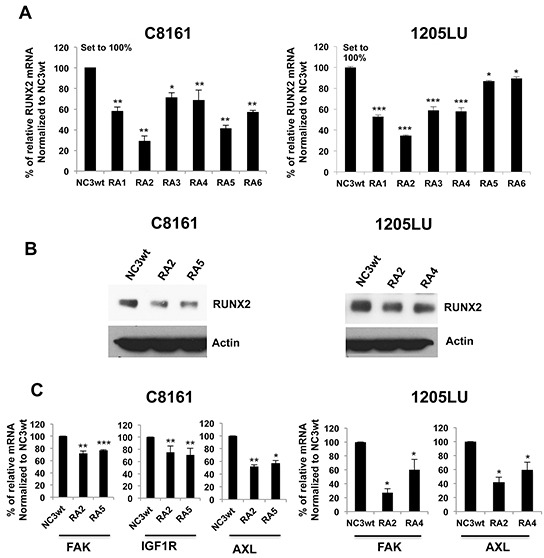
U1 Adaptor oligonucleotides targeting RUNX2 inhibit RUNX2 and RTK expression **A.** Expression of RUNX2 mRNA 72 hours after transfection of C8161 and 1205LU melanoma cell lines with the complexes, containing the dendrimer nanoparticle RGD-G5 and each of the 6 RUNX2 specific U1 Adaptors, named RA1, RA2, RA3, RA4, RA5 and RA6 (RA for RUNX2 Adaptor). This experiment, done in triplicate, is representative of three independent experiments. Results are expressed as % of relative RUNX2 mRNA normalized to the control NC3wt +/− SEM. **B.** Immunoblot analysis of RUNX2 expression 72 hours after transfection of C8161 and 1205LU melanoma cell lines with the complexes, containing the dendrimer nanoparticle RGD-G5 and two selected RUNX2 specific U1 Adaptors (RA2 and RA5 for C8161, RA2 and RA4 for 1205LU). **C.** Expression of FAK, IGF-1R and AXL 72 hours after transfection of C8161 cells with the RGD-G5-RA2 and RGD-G5-RA5 complexes and 72 hours after transfection of 1205LU cells with the RGD-G5-RA2 and RGD-G5-RA4 complexes. The samples were analyzed in quadruplicate. This experiment is representative of three independent experiments. Results are expressed as % of relative FAK, IGF1R or AXL mRNA normalized to the control NC3wt +/− SEM. * indicates p < 0.05, ** indicate p < 0.01 and *** indicate p < 0.001 compared with NC3wt based on Student's t-test.

To confirm the ShRNA data, we used a second and novel approach to target RUNX2 expression through a new strategy. This strategy uses U1 Adaptors, a recently discovered oligonucleotide-based-silencing technology whose unique mechanism of action targets nuclear pre-mRNA processing. A U1 Adaptor is a synthetic oligonucleotide (28-33 nucleotides) containing a 5′ target domain, which binds to the target pre-mRNA, and a 3′ U1 domain that binds to the 5′ end of the U1 small nuclear RNA subunit of U1 snRNP. As a result of the tethering of the U1 snRNP to the target pre-mRNA, maturation is prevented and reduced levels of mature mRNA are produced [51]. To deliver the U1 Adaptors to the cells, we used a tumor-targeting dendrimer nanoparticle previously described [52]. The dendrimer nanoparticle, known as RGD-G5, contains the cyclic RGD pentapeptide, a tumor targeting ligand, which specifically binds the α5β3 splice variant of an integrin cell surface receptor overexpressed in a wide variety of cancer cells. The RGD targeting ligand was coupled to the generation 5 (G5) polypropyleneimine (PPI) dendrimer in a final 2:1 molar ratio to give RGD-G5. Previous studies showed that RGD-G5 was active as a delivery vehicle [52]. Using the RGD-G5 dendrimer to deliver the U1 Adaptors, we screened 6 RUNX2-specific U1 Adaptors for their ability to reduce levels of RUNX2 mRNA. For that purpose, C8161 and 1205LU cells were incubated in the presence of the preformed RGD-G5: RUNX2 U1 Adaptor complexes, for 72 hours, and RNA was then extracted. The 6 RUNX2 U1 Adaptors are designated RA1, RA2, RA3, RA4, RA5 and RA6 for RUNX2 Adaptors 1 through 6. The control used is designated NC3wt, a U1 Adaptor, which does not target any human gene, but can still bind U1 snRNP through its U1 domain. As shown in Figure 3A, real time PCR determined that RA2 Adaptor was the most efficient in reducing *RUNX2* mRNA levels in both C8161 and 1205LU melanoma cells, lowering *RUNX2* mRNA levels to less than 40% of those in the presence of the control NC3wt. For C8161, RA5 and RA6 were efficient in reducing to 40 and 60% of the control respectively. For 1205LU, RA1, RA3 and RA4 decreased RUNX2 levels to about 60% of the control. For the next experiments, we used RA2 and RA5 Adaptors for C8161 cells and RA2 and RA4 Adaptors for 1205LU cells. As shown in Figure 3B, RA2 and RA5 for C8161 cells and RA2 and RA4 for 1205LU cells decreased RUNX2 protein expression.

In order to determine whether decreased RUNX2 expression translated into reduced RUNX2 activity and decreased RTK expression, we analyzed the expression of FAK, IGF1R and AXL mRNA by qPCR after transfection of C8161 cells with RA2 or RA5 Adaptors and 1205LU cells with RA2 or RA4 Adaptors. We previously showed that RUNX2 regulates the expression of the FAK protein in melanoma cells [14]. Therefore, we used *FAK* mRNA as a control for RUNX2 loss of activity and confirmed a reduction of *FAK* expression in melanoma cells transfected with the selected Adaptors RA2 and RA5 for C8161 cells, and RA2 and RA4 for 1205LU cells (Figure 3C). In addition, these results suggest that the effect of RUNX2 on FAK occurs at the transcriptional level. We further analyzed the effect of RUNX2 knock down on the mRNA expression of two previously identified RTKs, *IGF-1R* and *AXL*. Figure 3C shows that RA2 and RA5 for C8161 and RA2 and RA4 for 1205LU decrease *AXL* mRNA expression, demonstrating that the effect of RUNX2 on AXL protein expression (Figure 1B) occurs at the transcriptional level. We also found that RA2 and RA5 decrease *IGF-1R* mRNA expression in C8161 melanoma cells (Figure 3C) in contrast to RA2 and RA4, which do not inhibit *IGF1R* mRNA expression in 1205LU melanoma cells (data not shown). It is possible that the effect of RUNX2 on IGF1R protein expression (Figure 1B) does not occur at the transcriptional effect in 1205LU melanoma cells. Alternatively, it is also conceivable that a complete knock down of RUNX2 has to be achieved in order to see an effect on *IGF1R* mRNA expression in 1205LU cells as suggested by Figure 1B. Altogether, these studies demonstrate that RUNX2-specific U1 Adaptors significantly decrease the mRNA and protein expression of RUNX2 and mRNA expression of some of its targets genes, *FAK, AXL and IGF1R*.

### AKT activity is involved in RUNX2 and RTK expression

**Figure 4:**
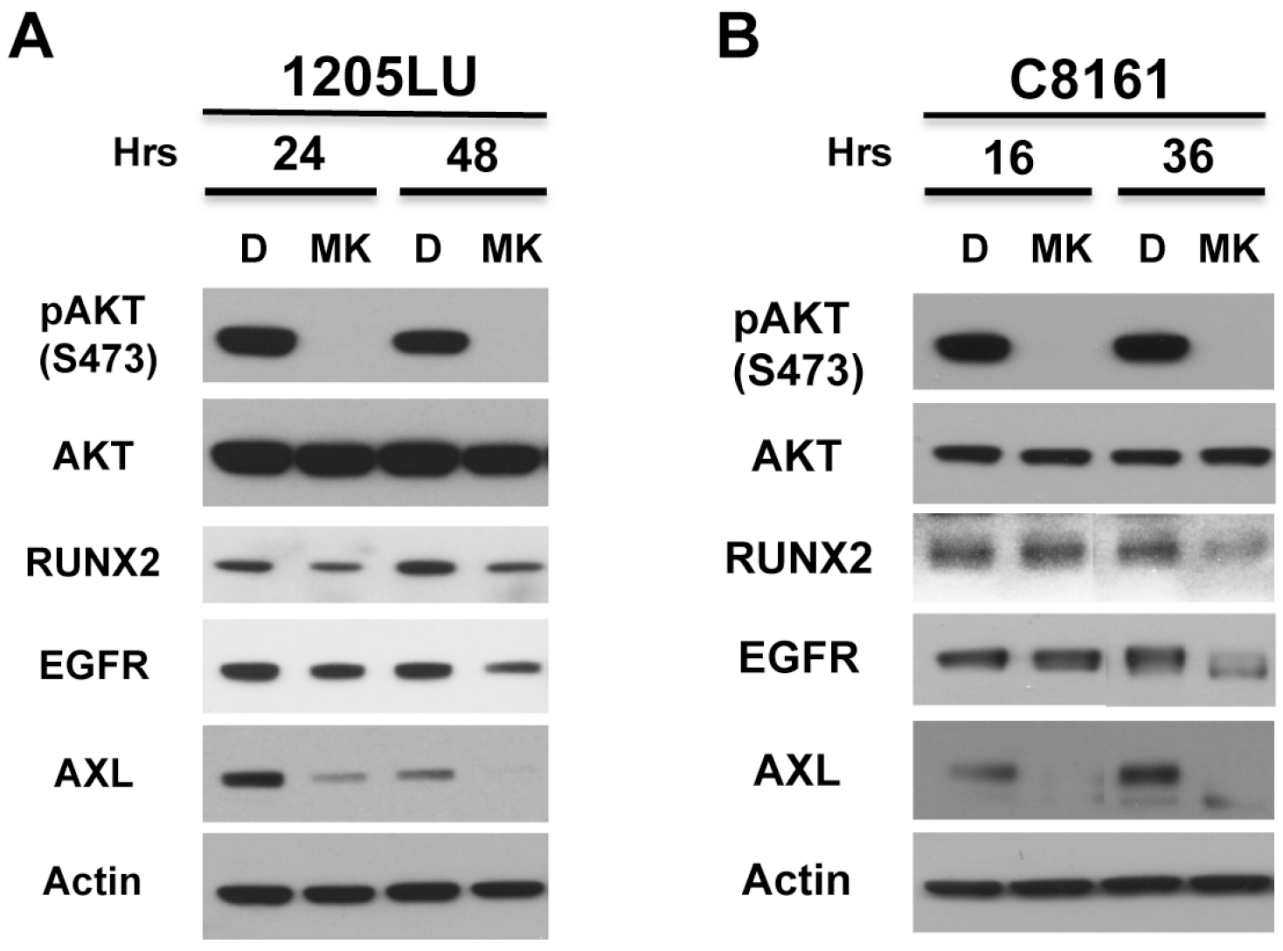
AKT activity is involved in RUNX2 and RTK expression 1205LU **A.** and C8161 **B.** melanoma cell lines were treated with vehicle (DMSO, (D)) or 10 μM MK2206 (MK) for the indicated times. Lysates were analyzed for the expression of pAKT, AKT, RUNX2, EGFR, AXL and Actin.

1205LU cells expressing ShRUNX2-3 and exhibiting a decrease in RUNX2, IGF-1R, EGFR and PDGFRβ levels also demonstrated a reduction in pAKT2 (S474) and pAKT (T308) levels. This suggests that RUNX2 might play a role in maintaining activation of the PI3K/AKT pathway through the regulation of RTKs in some melanoma cells. Furthermore, several studies suggested a functional cooperation of RUNX2 and the PI3K/AKT pathway as a driving force for tumor progression in different cancer types [8]. This functional interaction led us to hypothesize that AKT activity could also play a role in maintaining RUNX2 levels in melanoma cells. To test this hypothesis we used MK2206, an allosteric AKT inhibitor [53]. 1205LU and C8161 melanoma cells were treated with 10 μM MK2206 for 24 and 48 hours or for 16 and 36 hours respectively. As shown in Figures 4A and 4B, treatment with MK2206 decreased levels of pAKT (S473) as expected for both lines while levels of AKT were unchanged. 1205LU melanoma cells exhibited reduced levels of RUNX2 expression at the two time points and a parallel decrease in EGFR and AXL expression. C8161 exhibited a decrease in RUNX2 expression at the 36-hour time point, with a parallel decrease in EGFR and AXL expression. At the 16-hour time point, C8161 cells showed a decrease in AXL expression while RUNX2 levels were unchanged. This data suggests that MK2206 effect on AXL expression could be RUNX2 dependent or RUNX2 independent.

### RUNX2 isoforms 1, 2 and 3 are expressed at varying levels in melanoma cells

**Figure 5:**
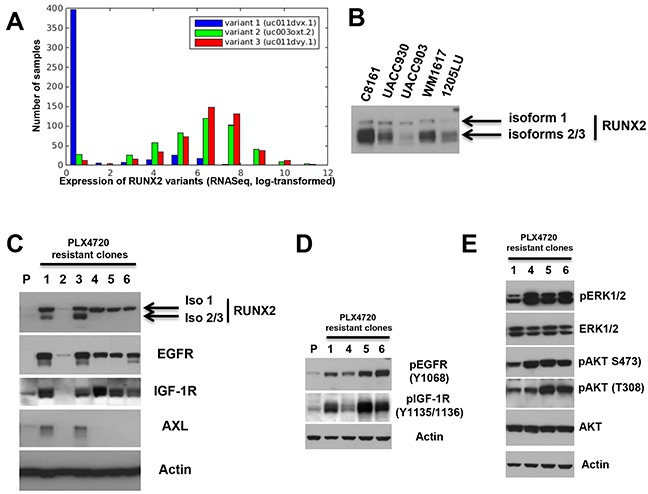
Increased expression of RUNX2 isoforms and RTKs in melanoma cells resistant to BRAF V600E inhibition **A.** An isoform level analysis of RUNX2 expression in 471 samples from the TCGA-SKCM (Skin Cutaneous Melanoma) cohort shows expression of variants 2 and 3 in the cohort, while variant 1 is mostly absent. Shown is the distribution of log-transformed expression values for each isoform (X axis) across the samples. Y axis: Number of samples. **B.** Expression of RUNX2 isoforms 1, 2 and 3 in melanoma cell lines. **C.** Clones resistant to 3 μM (clones 1-3), 5μM ((clone 4) and 0.5 μM (clones 5 and 6) PLX4720 were analyzed for RUNX2, EGFR, IGF-1R and AXL expression. P: Parental 1205LU melanoma cell line. D. Levels of pEGFR (Y1068) and pIGF-1R (Y1135/1136) in parental 1205LU melanoma cells (P) and clones 1, 4, 5 and 6 resistant to PLX4720. E. Levels of pERK1/2, pAKT (pan) (S473), pAKT (T308), ERK1/2 and AKT in clones 1, 4, 5 and 6 resistant to PLX4720.

We previously published the expression of RUNX2 in melanoma cell lines and RUNX2 appeared as a single band on low exposure [14]. However, three main RUNX2 isoforms are produced through alternative splicing. Isoform 1 (identifier Q13950-1) is the chosen canonical sequence and the longest isoform. Isoform 2 (identifier Q13950-2) has a different N terminal domain as compared with isoform 1: 1-19: MASNSLFSTVTPCQQNFFW → MRIPV. Isoform 2 is shorter than isoform 1. Isoform 3 (identifier Q13950-3) differs from isoform 1 by an internal deletion for which amino acids 341-362 are missing. To analyze the presence and expression of RUNX2 isoforms in human tumors, we downloaded isoform expression data from 471 samples from the TCGA-SKCM cohort. Figure 5A shows the distribution of normalized expression values of the three isoforms in this set (histogram, log-transformed expression values on the x-axis). Isoforms 2 and 3 are expressed in the cohort, while isoform 1 is mostly not present. Isoforms 2 and 3 are expressed together with a correlation of 0.79 (p<1.0e-100). Using a monoclonal antibody raised against a synthetic peptide surrounding A273 of human RUNX2 we were able to detect the three isoforms, as shown in Figure 5B. In accordance with the TCGA data analysis, isoform 1 is expressed at lower level than isoforms 2 and 3, which often appear together as a thicker band (Figure 5B). Experiments presented on Figures 1, 3 and 4 mainly showed isoforms 2 and/or 3 because of the low expression of isoform 1 as compared with the other two isoforms. The experiments presented in Figure 5C (see below) show the appearance of isoform 1 at higher levels.

### Increased expression of RUNX2 isoforms in melanoma cells resistant to BRAF V600E inhibition

The up-regulation of EGF-R, PDGFRβ, AXL and IGF-1R expression/activity plays a crucial role in resistance mechanisms to targeted therapies using BRAF V600E inhibitors [19–22, 24, 25]. Since we showed co-regulation of RUNX2 and those RTKs, we postulated that resistance to BRAF V600E inhibition could be associated with an increase in RUNX2 expression. In the first set of experiments, 1205LU melanoma cells were treated with the BRAF V600E inhibitor PLX4720 *in vitro* and clones resistant to PLX4720 were selected and expanded. Two clones resistant to 0.5 μM, three clones to 3 μM and one clone to 5 μM PLX4720 were analyzed for RUNX2 expression. As shown in Figure 5C (low exposure), two out of three clones resistant to 3 μM PLX4720 (clones 1 and 3), exhibited increased level of RUNX2 isoforms 2/3, while an increase in isoform 1 was observed in all but one clone resistant to PLX4720. All the clones expressing RUNX2 isoform 1 showed increased levels of EGFR and IGF-1R, while we found increased AXL expression in clones 1 and 3 up-regulating RUNX2 isoforms 2/3 in addition to isoform 1. We further analyzed four of the six 1205LU clones resistant to PLX4720 (clones 1,4, 5 and 6) for the levels of phosphorylated/activated EGFR and IGF-1R. We demonstrate increased expression of pEGFR (Y1068) and pIGF-1R (Y1135/1136) in the four clones as compared with the parental 1205LU melanoma cells (Figure 5D). In addition, we show constitutive ERK1/2 and AKT (S473 and T308) phosphorylation in those four PLX4720 resistant clones (Figure 5E). Therefore, our results show an increase in RUNX2 levels and an associated increase in RTK levels and activation in 1205LU clones resistant to PLX4720.

**Figure 6:**
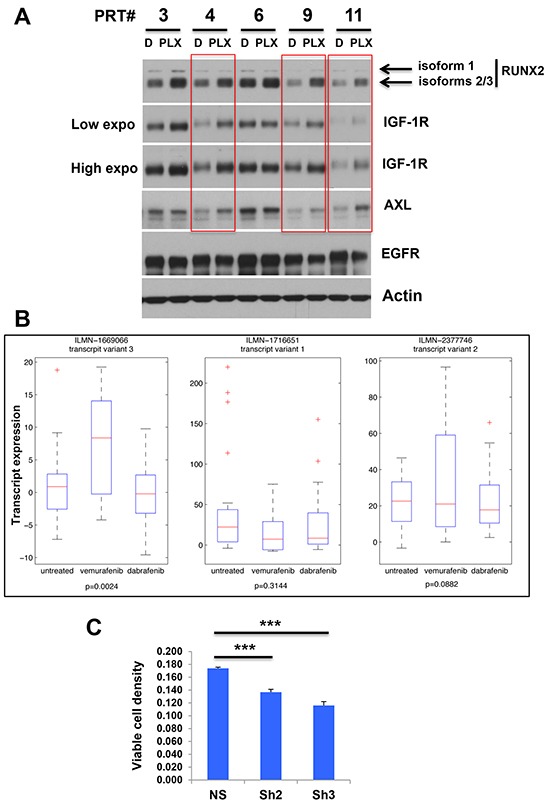
**A.** Expression of RUNX2 and RTKs in melanoma cells rendered resistant to BRAF V600E inhibition *in vivo*. Melanoma cell lines established from PLX4720-resistant 1205LU xenografts (PRT lines 3, 4, 6, 9 and 11) were incubated in the presence of DMSO (D) or 1 μM PLX4720 (PLX) for 24 hours and RUNX2, AXL, IGF-1R and EGFR expression was analyzed. Low and high exposures for IGF1R are shown. **B.** Patient data from a cohort containing samples from untreated tumors and tumors treated with Vemurafenib and Dabrafenib respectively [56] were downloaded from GSE50509. Expression of RUNX2 transcripts 1, 2 and 3 in untreated and Vemurafenib and Dabrafenib treated groups. P-values were calculated using a t-test between untreated and vemurafenib treated groups.” **C.** 1205LU melanoma cells expressing non-silencing ShRNA (NS), ShRUNX2-2 (Sh2) or ShRUNX2-3 (Sh3) were treated with Vehicle (DMSO) or 10 μM PLX 4720 for 72 hours and viable cells were then counted. This experiment, done in triplicate, is representative of three independent experiments. Results are expressed as % (cell numbers in the presence of PLX4720/cell numbers in the presence of DMSO). *** indicate p < 0.001 compared with NS.

In the second set of experiments we took advantage of the existence of cell lines established from PLX4720-resistant tumors (PRT) in Dr. A. Aplin's laboratory (Kimmel Cancer Center Philadelphia, PA) [54, 55]. Briefly, 1205LU xenograft tumors initially shrank in the presence of PLX4720 and rapid regrowth occurred associated with ERK1/2 reactivation [54, 55]. Five cell lines established from the resistant tumors, PRT3, PRT4, PRT6, PRT9 and PRT11 were treated with vehicle or 1 μM PLX4720 for 24 hours *in vitro* as previously described [54]. As shown in Figure 6A, the expression of RUNX2 isoforms 2/3 was increased in the five PRT lines in the presence of PLX4720. An increase of isoform 1 was only observed in PLX4720-treated PRT3. These results suggest that PLX4720-resistant cells developed in an *in vivo* context can exhibit an increase in RUNX2 levels when reexposed to PLX4720 *in vitro*. These findings would also suggest that RUNX2 might be involved in acquired resistance. In parallel to the RUNX2 increase, we observed an increase in IGF-1R expression in four out of five PLX4720-treated PRT lines (PRT lines 3, 4, 9 and 11) and in AXL expression in three out of five PLX4720-treated PRT lines (PRT lines 4, 9 and 11). High endogenous EGFR expression in those PRT lines was not further modulated in the presence of PLX4720.

To address the relevance of these findings in human melanoma, patient data from a cohort containing samples from untreated tumors and tumors treated with vemurafenib and dabrafenib respectively [56] were downloaded from GSE50509. The data was analyzed on probe level in order to avoid low expressed variants to distort the evaluation. Probes for all three transcript variants were represented on the Illumina array. The expression of RUNX2 isoform 3 was significantly higher in vemurafenib treated patients compared to the untreated group (p=0.0024, Student's T-test). The expression of isoform 2 was slightly increased in this group (p=0.0882), while isoform 1 was not significantly changed among the groups (Figure 6B). These results showing the up-regulation of specific isoforms of RUNX2 in melanoma lesions from patients treated with Vemurafenib, suggest that chronic exposure to BRAF V600E inhibitors (PLX4720/Vemurafenib) may favor RUNX2 up-regulation and subsequent RTK up-regulation, an important player in acquired resistance to these drugs.

To determine whether RUNX2 expression played a role in sensitivity to PLX4720, we treated 1205LU melanoma cells expressing non-silencing ShRNA, ShRUNX2-2 or ShRUNX2-3 with 10 μM PLX4720 for 72 hours and counted viable cells. As shown in Figure 6C, RUNX2 knock down resulted in an increased sensitivity to PLX4720 as compared with control cells.

## DISCUSSION

**Figure 7:**
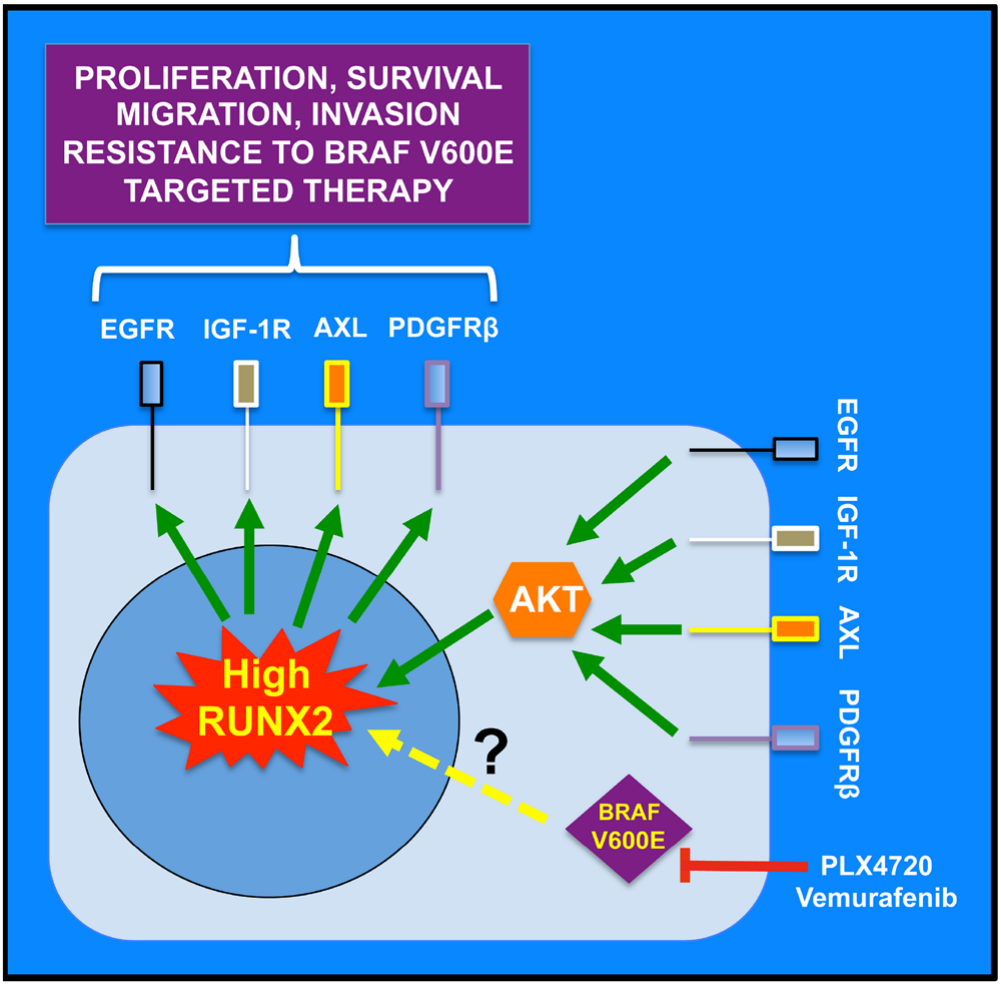
Working model for the role of RUNX2 in RTK-based autocrine loops and resistance to BRAF V600E targeted therapy RUNX2 regulates RTK expression by mechanisms yet to be defined. Treatment of melanoma cells with BRAF V600E inhibitors (PLX4720, Vemurafenib) eventually results in up-regulation of RUNX2 and subsequent up-regulation of RTKs, implicated in resistance to BRAF V600E targeted therapy, through increased proliferation, survival, migration and invasion. Reactivation of the oncogenic MAPK pathway through RTK up-regulation compensates for the inhibition of BRAF V600E. Those RTKs also signal through the PI3K/AKT pathway and AKT activation contributes to maintain high RUNX2 levels. This positive feedback loop contributes to drive progression of resistant melanoma cells.

In late stage melanomas, receptor tyrosine kinase-based autocrine loops contribute to the activation of pathways such as MAPK and PI3K/AKT [57]. However, little is known about the molecular events involved in the dysregulation of RTKs expression/activity. Our data identifies a candidate for this dysregulation. We initially demonstrated the over-expression of transcriptionally active RUNX2 in melanoma cell lines and melanoma samples as compared with primary melanocytes and melanocytic nevi respectively. We also implicated RUNX2 as an important factor in melanoma migration and invasion [14]. Our present study points to RUNX2 as a potential regulator of AXL, EGFR, IGF-1R and PDGFRβ and to a lesser extent FGFR1 expression. This conclusion is based on: 1) the down regulation of AXL, EGFR, IGF-1R and PDGFRβ in ShRUNX2 knocked down melanoma cell lines or RUNX2-specific U1 Adaptors-transfected melanoma cells. 2) The higher expression of FGFR1, EGFR and AXL in C8161 cells as compared with C81-61 cells, which parallels the higher level of expression of RUNX2 in C8161 cells; 3) the co-regulation of RUNX2 and AXL in a significant number of human melanoma samples (7 out of 39 samples with positive RUNX2 staining had strong AXL staining); 4) The increase in EGFR, IGF-1R and AXL expression in 1205LU clones resistant to the BRAF V600E inhibitor PLX4720 in association with dramatic RUNX2 up-regulation in 5 out of 6 clones analyzed; 5) The increase in IGF-1R and AXL expression in association with RUNX2 up-regulation in three out of five PLX4720-Resistant Tumor (PRT)-derived cell lines treated *in vitro* with PLX4720 as compared with vehicle-treated PRT cells. Furthermore, our results strongly suggest that RUNX2 might be a mediator of resistance to BRAF V600E targeted therapy, involving RTK up-regulation and activation in melanoma. A working model based on our findings is presented in Figure 7.

Because RUNX2 expression and activity are positively regulated by the PI3K and MAPK pathways [8], it is necessary to explore the relationship between IGF-1/IGF-1R and RUNX2. IGF-1 was shown to positively regulate RUNX2 expression in endothelial cells (EC) [13] and osteoblasts [58], and to stimulate RUNX2 activity in EC [59] and osteoblasts [60]. In EC, IGF-1 regulates RUNX2 DNA binding through sequential activation of the PI3K/Pak1 and ERK1/2 signaling cascade [59], but independently of AKT. Another mechanism of IGF-1-mediated increase in RUNX2 activity has been proposed in osteoblasts. The transcription factor FOXO1 physically interacts with RUNX2 in osteoblastic cells and in COS-7 cells and inhibits RUNX2 binding to its cognate site within the *osteocalcin* promoter. Upon IGF1/insulin binding to their receptors, activation of the PI3K/AKT pathway leads to phosphorylation and nuclear exclusion of FOXO1 and reactivation of RUNX2 [60]. We speculate that similar regulation of IGF-1 on RUNX2 expression and activity could exist in melanoma cells, through the activation of IGF-1R and subsequent stimulation of the PI3K and/or the MAPK pathways resulting in RUNX2 positive regulation. If such a system exists, the regulation of IGF-1R by RUNX2 shown in the present study would suggest the existence of a positive feedback loop of RUNX2/IGF-1/IGF-1R in melanoma cells, promoting melanoma migration and invasion. Similarly, the positive regulation of AXL, EGFR and PDGFRβ by RUNX2 could result in an activation of these receptors in the presence of their ligands (provided by melanoma cells or the melanoma cell microenvironment) thereby promoting stimulation of the PI3K and MAPK pathway and RUNX2 positive regulation (as shown in Figure 7).

AXL belongs to the TAM (Tyro3, AXL, MER) family of RTKs [61]. Real time PCR examination of 8 AXL-positive tumors demonstrated that *MER* and *TYRO3* transcripts were barely detectable and immunoblot analysis of representative melanoma cell lines confirmed mutual exclusion of AXL and TYRO3 and frequent co-expression of TYRO3 and MER [40]. Another study analyzed active RTKs in melanoma cell lines through measurement of their level of tyrosine phosphorylation and found that AXL, TYRO3 and MER were among the RTKs with the highest overall activation level. Co-activation of TYRO3 and MER was also frequently observed in these melanoma cell lines [35]. TYRO3 has been identified as an upstream regulator of the Microphthalmia-associated transcription factor (MITF). TYRO3 induces *MITF-M* expression in a SOX10-dependent manner in melanoma cells [62], while other studies showed the anti-correlation between MITF and AXL in melanoma cells [40, 41]. These results suggest that AXL and TYRO3 might play different roles in melanoma progression, perhaps in relationship with MITF expression. Interestingly, eighteen AML1a (RUNX1) binding sites have been predicted to exist in the TYRO3 promoter, according to DECODE (DECipherment Of DNA Elements), a SABiosciences’ proprietary database combining their Text Mining Application and data from the UCSC Genome Browser. Therefore, a parallel can be drawn between the regulation of AXL by RUNX2 and the potential regulation of TYRO3 by RUNX1. However, more studies are required to confirm the regulation of TYRO3 by RUNX1, and to decipher the role of RUNX1 in melanoma cells, which is co-regulated with RUNX2 with a Pearson's Correlation of 0.31 in cutaneous melanoma (TCGA provisional, 278 samples) using CBio Portal (http://www.cbioportal.org) [43, 44].

We show that when the five PRT lines derived from the PLX4720-resistant 1205LU melanoma xenografts are treated *in vitro* with PLX4720, all PRT lines exhibit an increased RUNX2 expression (isoforms 2/3) as compared with vehicle-treated cells. In addition, we demonstrate an increase in IGF-1R expression in four out of the five PLX4720-treated PRT lines, while AXL expression is increased in three out of five PLX4720-treated PRT lines. PRT lines 4, 9 and 11 show parallel increase in RUNX2, IGF-1R and AXL (Figure 6A). The selective increase in RUNX2 isoforms 2/3 observed in PLX4720-treated PRT lines derived from PLX4720-resistant tumors (Figure 6A) and in Vemurafenib-treated patients (Figure 6B) is in apparent contradiction with the high expression of isoform 1 in all but one 1205LU clones developing resistance to PLX4720 *in vitro* (Figure 5C). In 1205LU clones developing resistance *in vitro*, two of the six clones expressed increased levels of isoforms 2/3. The tumor microenvironment present during the *in vivo* treatment with the BRAF V600E inhibitors (PLX4720-treated xenografts and Vemurafenib-treated patient tumors) but absent in clones developing PLX4720 resistance *in vitro*, likely plays a role in these differences in the type of RUNX2 isoforms up-regulated.

Interestingly, PRT6 was shown to carry a HRAS Q61K mutation; PRT3 was shown to express a previously unreported variant that splices exon 2 with exon 11 of BRAF V600E; PRT4 was found to express a variant previously reported from a patient sample that splices exon 1 with exon 9 of BRAF V600E. Both PRT3 and PRT4 maintained V600E positivity [54]. The BRAF V600E variants and the HRAS mutation were sufficient for resistance to PLX4720 treatment. This study illustrates that from a single cell line, multiple mechanisms of resistance could emerge [54]. Due to the heterogeneity of resistant tumors, these authors proposed taking several biopsies from multiple sites for molecular analysis [54]. Our data suggests that in addition to examining RAS mutations and BRAF variants during the molecular analysis of tumor samples, we should also include the analysis of RTK up-regulation/activation, as it participates in a significant number of acquired resistance mechanisms, possibly in conjunction with RAS mutation or the existence of V600E splicing variants.

Regarding resistance to RTK inhibition, we observed that downregulation of IGF-1R and AXL in C8161 melanoma cells expressing ShRUNX2-3 (Figure 1B) did not result in reduced AKT and ERK phosphorylation (Figure 1C) but instead increased AKT phosphorylation. It is possible that the increased activation of EGFR whose expression was unchanged or expression/activation of another RTK compensated for the reduction in IGF-1R- and AXL-mediated signaling. Such a compensatory mechanism has been described in non-small cell lung cancer (NSCLC) cells: Treatment with the EGFR erlotinib inhibitor induced heterodimerization of insulin-like growth factor receptor/epidermal growth factor receptor, activated IGF-1R and downstream signaling mediators, which ultimately counteracted the antitumor action of erlotinib in NSCLC cells [63]. These results support the hypothesis that the reactivation of other RTKs (through homo or heterodimerization) is likely to happen following down regulation of one or two RTKs or their inhibition by small molecules inhibitors.

We demonstrated that RUNX2 knock down resulted in down regulation of FAK ([14] and Figure 3) in addition to IGF-1R, EGFR, PDGFRβ and AXL decrease. Interestingly, disruption of the protein interaction between FAK and IGF-1R by a small molecule (INT2-31) induced apoptosis and cell cycle arrest, inhibited AKT phosphorylation *in vitro* and in tumors, and decreased growth of melanoma xenografts [37]. These results suggest that targeting RUNX2 expression or activity might induce similar effects by negatively affecting FAK-IGF-1R interaction. In addition, the involvement of RUNX2 in the regulation of four different RTKs implicated in melanoma progression and acquired resistance to BRAF V600E inhibitors suggests that RUNX2 is a potential therapeutic target in patients with melanoma. RUNX2 belongs to a class of proteins traditionally considered undruggable. However, transcription factors have become the focus of new targeting strategies: These new strategies exploit the fact that transcription factor activity is regulated at distinct levels and that we can interfere with each of these levels, including specific binding to cis-regulatory elements or homo- and hetero-dimerization [64, 65]. There is increasing evidence that transcription factors play oncogenic roles in melanoma and this has driven efforts to develop new approaches to target this class of proteins [66]. Targeting RUNX2 binding to cis-regulatory elements and RUNX2 interaction with major co-activators/transcription partners, such as SMADS [8, 67] are likely to open new avenues of therapy for patients with melanoma.

## MATERIALS AND METHODS

### Cell lines

WM9, WM1617, WM793, WM278, and 1205LU were kindly provided by Dr. M. Herlyn (Wistar Institute, Philadelphia, PA, USA [49]). These lines were cultured in MCDB153/L-15 (4/1 ratio) medium containing 2% FBS, 5 μg/ml Insulin and 1.7 mM Calcium Chloride. C8161 and C81-61 melanoma cell lines were provided by Dr. Mary Hendrix (Children's Memorial Research Center, Chicago, IL, USA [50] and were grown in D-MEM (Mediatech, 10-013-CV) containing 10% FBS. UACC903 and UACC930 cells were provided by Dr. Jeffrey M. Trent (Translational Genomics Research Center, Phoenix, AZ, USA [68]) and were grown in RPMI1640 (Invitrogen, 11875) containing 10% FBS. WM115 and WM35 melanoma cell lines were purchased from ATCC (American Type Culture Collection, Manassas, VA 20110, U.S.A). WM35 and WM115 [49] were grown in MCDB153/L-15 (4/1 ratio) medium containing 2% FBS, 5 μg/ml Insulin and 1.7 mM Calcium Chloride. PLX4720 resistant clones were kindly provided by Dr S. Chen (Rutgers University, Piscataway NJ). Briefly, 2-4 × 10^5^ 1205LU melanoma cells were plated in a 60 mm dish in the presence of increasing concentrations of PLX4720 (from 0.1 to 5 μM). Resistant clones were then expanded successively in 24-well plates, 35 and 60 mm dishes in the presence of the respective concentrations of PLX4720.

### Treatment with pharmacological agents

For MK2206 treatment, 1205LU and C8161 melanoma cell lines were treated with vehicle (DMSO) or the allosteric AKT inhibitor, MK2206 (10 μM), for 24 and 48 hours and for 16 and 36 hours respectively. Whole cell lysates were then prepared as described below in the immunoblotting section. For PLX4720 treatment of 1205LU melanoma cells expressing non-silencing ShRNA, shRUNX2-2, or ShRUNX2-3, 10^5^ cells were seeded, treated the next day with 10 μM PLX4720 for 72 hours and then counted using a Vi-Cell counter. For PLX4720 treatment of the PRT lines, 5×10^5^ PRT (PLX4720-Resistant Tumors) -derived cell lines (PRT lines 3, 4, 6, 9 and 11 [54]) were seeded on a 10 cm plate and allowed to proliferate without drug for 72 hrs. Then cells were then treated with vehicle (DMSO) or 1 μM PLX4720 for 24 hrs, before protein extraction.

### Proteomics analysis of ShRNA RUNX2-expressing melanoma cells using iTRAQ protocol

Samples were lysed as previously described [14]. The protein samples (120 ug) were run into a Novex Bis-Tris 10% gel as gel plugs and digested with trypsin using standard protocol. The digested peptides were washed with methanol and labeled with iTRAQ reagent (Sciex) using manufacturer's recommended protocol and then combined.

High-pH Reverse phase HPLCs were used for peptide fractionation using Gilson 300 series. Samples were solubilized in 200 μl of 20 mM ammonium formate (pH10), and injected onto an Xbridge column (Waters, C18 3.5 μm 2.1X150 mm) using a linear gradient of 1%B/min from 2-45% of B (buffer A: 20 mM ammonium formate, pH 10, B: 20 mM ammonium formate in 90% acetonitrile, pH10). 1min fractions were collected and speed vac dried.

Selected fractions were either combined or directly analyzed by LC-MS/MS. 5μl/12.5 μl of fractionated samples were analyzed by nanoLC-MS/MS using a RSLC system (Dionex, Sunnyvale CA) interfaced with a LTQ Orbitrap Velos (ThermoFisher, San Jose, CA). Samples were loaded onto a self-packed 100μm x 2cm trap packed with Magic C18AQ, 5μm 200 A (Michrom Bioresources Inc, Aubum, CA) and washed with Buffer A (0.2% formic acid) for 5 min with a flow rate of 10ul/min. The trap was brought in-line with the homemade analytical column (Magic C18AQ, 3μm 200 A, 75 μm x 50cm) and peptides fractionated at 300 nL/min with a multi-stepped gradient (4 to 15% Buffer B (0.16% formic acid 80% acetonitrile) in 25 min and 15-25%B in 65 min and 25-50%B in 55 min). Mass spectrometry data was acquired using a data-dependent acquisition procedure with a cyclic series of a full scan acquired in Orbitrap with resolution of 60,000 followed by MSMS scans (HCD 38% of collision energy) of 10 most intense ions with a repeat count of two and the dynamic exclusion duration of 60 sec.

The LC-MSMS data was searched against the *human* Ensembl database using an in house version of X!tendem (SLEDGEHAMMER (2013.09.01), thegpm.org) with carbamidomethylation on cysteine and iTRAQ labeling on lysine and N-terminus of the peptide as fixed modification and oxidation of methionine as variable modification using a 10 ppm precursor ion tolerance and a 20 ppm fragment ion tolerance. Intensity of iTRAQ reporter ions of each spectrum was extracted using an in-house perl script corrected for isotope cross-over using values supplied by the manufacturer. The ratio was normalized using median intensity ratio of all identified spectra that fit certain criteria: peptide log€ <-1.5, sum of reporter ion intensity >4,000. Protein ratios were calculated using median value of ratios of all peptides belonging to the same protein that fits criteria described above.

### Immunoblotting

Cells were harvested, washed with PBS, and lysed with cell lysis buffer in the presence of protease and phosphatase inhibitors (Roche) as previously described [14]. Equal amounts of protein were separated on polyacrylamide gel electrophoresis and transferred onto nitrocellulose membrane, and immunoblots were analyzed using antibodies against RUNX2, GAPDH, FAK, EGFR, PDGFRβ, FGFR1, IGF-1R, pAKT1 (S473), pAKT2 (S474), pAKT (T308), phosphop44/42 MAPK (ERK1/2) (T202/Y204), pEGFR (Y1068), pAXL (Y702) from Cell Signaling (Danvers, MA), AXL from R&D systems (Minneapolis, MN), pIGF-1R (Y1135/1136) from ThermoFisher Scientific (Rockford, IL) and Actin (Sigma Aldrich, St. Louis, MO).

### Detection of AXL by immunohistochemistry

The human melanoma tissue microarray (TMA number ME1004A) was purchased from US Biomax, Inc. (Rockville, MD, USA). The melanoma TMA was deparaffinized and antigen retrieval was performed using extended CC1 treatment (Cell Conditioning Solution, Ventana Medical Systems, Oro Valley, AZ). The goat polyclonal antibody for AXL (R&D) was applied and incubated at 37°C for 1 or 2 hours. Donkey anti-goat secondary antibody (Jackson ImmunoResearch Laboratories, West Grove, PA) was then applied and incubated at 37°C for 60 min, followed by chromogenic detection using the DAB Map kit (Ventana Medical Systems, Oro Valley, AZ). Slides were counterstained with Hematoxylin and dehydrated and cleared before coverslipping from Xylene. RUNX2 staining was done as previously described [14].

### Analysis of IHC staining

Tissue microarray specimens were imaged using Trestle® whole slide imaging system under a 20x objective. Custom whole slide visualization software [69] developed at Center for Biomedical Imaging & Informatics, Rutgers Cancer Institute of Pathology, was used to display high-resolution, synchronized, side-by-side views of corresponding TMA cores stained with RUNX2 and AXL and a board-certified pathologist examined tissue composition and staining intensity at each TMA core location. Quantification of RUNX2 expression was previously reported [14]. Due to the presence of non-melanoma cells staining and melanin in the specimen, evaluation of AXL expression level was conducted in a semi-quantitative manner. A board-certified pathologist closely examined side-by-side views of each AXL stained TMA core with its RUNX2 staining counterpart, and determined co-localizing AXL expression level in melanoma cells to be “Strong”, “Faint” or “Negative”.

### RUNX2 knock down using ShRNA

We used two different human RUNX2 ShRNA, targeting either the coding sequence (ShRUNX2-3) or the 3′UTR (ShRUNX2-2), in the pGIPZ lentiviral vector. The mature senses were CCAGCTGCATCCTATTTAA for ShRUNX2-2 and ACAAGGACAGAGTCAGATT for ShRUNX2-3. The mature sense was ATCTCGCTTGGGCGAGAGTAAG for the non-silencing control. This sequence does not match any known mammalian gene (has at least 3 or more mismatches against any gene as determined via nucleotide alignment/BLAST of 22mer sense sequence). 80-90 % confluent 293 amphotropic cells were transfected with 10 μg of non-silencing control or shRUNX2 plasmid and 4 μg of PREV (pcmv-dR8.2 dvpr) plasmid using Lipofectamine 2000 (Invitrogen, Carlsbad, CA). 48 hours following transfection, the supernatant media was filtered through a 0.4uM filter for infection. Melanoma cell lines C8161, WM35 and 1205LU were seeded at a density of 0.2 × 10^6^ cells/6 well plate 24 hours prior to the infection. The cells were then infected with 1 ml viral particles and 8ug/ml polybrene and after 6 hours, 1ml fresh media was added and incubated for overnight. The following day, cells were incubated with 2 ml fresh media followed by stable selection with 3 μg/ml Puromycin for 1 to 2 weeks.

### RUNX2 mRNA targeting using anti-RUNX2 U1 Adaptors

The panel of anti-RUNX2 U1 Adaptors was designed by and then purchased from Silagene Inc. (Hillsborough, NJ). The RGD-G5 dendrimer was prepared as previously described [52]. The 1205LU and C8161 cells were plated at 200,000 cells per well in a 6 well plate. Transfection of anti-RUNX2 U1 Adaptors or control U1 Adaptors in complex with RGD-G5 dendrimers was done as follows: for a 6-well plate, a 0.2 ml transfection mix containing 20mM HEPES buffer pH 7.4 in water was prepared containing 200nM U1 Adaptor and 1500nM RGD-G5 dendrimer, and the solution gently mixed. The RGD-G5:anti-RUNX2 U1 Adaptors complexes were then added to cells that had been overlaid with 1.8 ml of fresh growth media giving a final concentration of 20nM U1 Adaptor and 150nM RGD-G5 dendrimer. After 72 h, either total RNA was extracted for qPCR or protein extracted for Western blotting.

### RNA extraction, RT-PCR and real-time PCR for the experiments with U1 Adaptors

Total RNA was extracted from transfected cells using Trizol reagent (Invitrogen, Carlsbad, CA) and further purified using NucleoSpin RNA kit (Macherey-Nagel, Germany) according to the manufacturer's instructions. 500 ng of total RNA was used for cDNA synthesis with random hexamers using high capacity cDNA reverse transcription kit (Applied Biosystems) according to the manufacturer's instructions. For RT-PCR amplifications SYBR Green PCR master mix (Applied Biosystems) was prepared with appropriate forward and reverse primers and cDNA according to the manufacturer's instructions. The thermal cycling conditions were composed of an initial denaturation step at 95°C for 10 min, 40 cycles at 95°C for 30s, 55°C for 30s and 72°C for 30s with a Mx3000P (stratagene). A melting curve was generated by slowly increasing (0.1°C/s) the temperature from 60°C to 95°C, while the fluorescence was measured. Reactions were run in duplicates in three independent experiments. The housekeeping gene GAPDH was used as an internal control for normalization. The fold change in the mRNA levels in the RUNX2 U1 Adaptor-transfected cells was analyzed by comparing to RGD-G5 transfected cells using the 2 -ΔΔCT method previously described [70]. The results are presented as percentage of RUNX2 remaining by setting the value from RGD-G5 transfected cells to 100%. Data from qPCR experiments are presented as the average ± standard error of the mean. Statistical analysis was performed by Student's t test and *P* values < 0.05 were considered statistically significant.

### Patient data analysis

Level 3 RNASeqV2 isoform expression data from 471 samples in the TCGA-SKCM cohort were downloaded and RUNX2 isoform probes corresponding to the three variants were selected. The rsem normalized values were log-transformed (log2(data+1)) and a histogram was generated using the MATLAB routine *hist*.

Patient data from a cohort containing samples from untreated tumors and tumors treated with vemurafenib and dabrafenib respectively [56] were downloaded from GSE50509. The data was downloaded in the pre-normalized form, and probes for RUNX2 were selected. We used the box plot routine (MATLAB) to plot the three different treatment groups for all probes individually. The plot shows data between 25th and 75th percentile within the blue boxes and the median as red line in each group. Whiskers indicate the 5th and 95th percentiles and outliers are indicated as data points in red. P-values were calculated using a t-test between untreated and vemurafenib treated groups.”
